# Visualizing Risk Prediction Models

**DOI:** 10.1371/journal.pone.0132614

**Published:** 2015-07-15

**Authors:** Vanya Van Belle, Ben Van Calster

**Affiliations:** 1 KU Leuven, Department of Electrical Engineering (ESAT), STADIUS Center for Dynamical Systems, Signal Processing and Data Analytics, Leuven, Belgium; 2 iMinds Medical IT, KU Leuven, Leuven, Belgium; 3 KU Leuven, Department of Development and Regeneration, Leuven, Belgium; University of Connecticut, UNITED STATES

## Abstract

**Objective:**

Risk prediction models can assist clinicians in making decisions. To boost the uptake of these models in clinical practice, it is important that end-users understand how the model works and can efficiently communicate its results. We introduce novel methods for interpretable model visualization.

**Methods:**

The proposed visualization techniques are applied to two prediction models from the Framingham Heart Study for the prediction of intermittent claudication and stroke after atrial fibrillation. We represent models using color bars, and visualize the risk estimation process for a specific patient using patient-specific contribution charts.

**Results:**

The color-based model representations provide users with an attractive tool to instantly gauge the relative importance of the predictors. The patient-specific representations allow users to understand the relative contribution of each predictor to the patient’s estimated risk, potentially providing insightful information on which to base further patient management. Extensions towards non-linear models and interactions are illustrated on an artificial dataset.

**Conclusion:**

The proposed methods summarize risk prediction models and risk predictions for specific patients in an alternative way. These representations may facilitate communication between clinicians and patients.

## Introduction

The focus on risk prediction models is increasing in many areas of clinical research. These tools can aid doctors in personalized decision-making regarding diagnosis, prognosis and treatment. Examples are the Nottingham Prognostic Index to allocate breast cancer patients to risk groups, the Apache system to predict the risk of hospital mortality, the IOTA LR2 model to estimate the risk of malignancy of ovarian tumors and the euroSCORE to calculate the predicted operative mortality for patients undergoing cardiac surgery [1–5]. However, prediction models do not easily find their way to routine clinical practice. Practical obstacles are one issue: ideally the models should seamlessly fit the clinician’s workflow, e.g. by being computerized, automatic, and built in into electronic health records [6–8]. Another, sometimes undervalued issue is that of clinical credibility [9]. One aspect of credibility is to have understanding of how a model arrives at an estimated risk [10]. As a result, model representation and visualization are essential issues in prediction modeling research. This is further strengthened by the rise of shared decision-making and patient centeredness [11,12].

In many, if not most, risk prediction studies, details are given on aspects including the choice of a suitable underlying model, the selection of relevant predictors, independent evaluation of the models, discrimination and calibration performance [13–16]. Unfortunately, the choice of the representation of the developed models for interpretation and practical use is often overlooked [17], although this presentation is crucial in the relation between doctor and patient [18]. Research articles typically represent a prediction model by means of the estimated coefficients or a table with odds ratios or hazard ratios. However, interpreting these values is non-intuitive and depends on the unit of the predictors [19–22]. Direct comparisons of coefficients and odds ratios for different predictors is therefore difficult in a clinical setting [20–23]. In addition, risk prediction models are often implemented in software tools that provide risk estimates when predictor values are entered [24,25,26]. Although such implementations are vital, the interpretability of the original model is lost. There is no information on how the model arrived at the risk estimate by indicating which predictors contributed most. Hence, for prognostic applications including predictors that can be influenced by the patient, the model gives no direct insight into how the estimated risk could be lowered through changes in lifestyle or medication.

To address this issue, different alternative model representations have been proposed. Representing a logistic regression model or a Cox model by means of a nomogram [27,28] is popular in certain areas. Alternatively, regression models can be approximated by means of score systems [15,29]. The advantage of these systems is that a number of points is allocated to each predictor value, with higher values indicating a larger contribution to the risk estimate. A disadvantage is that there are no guidelines to choose the predictors values at which the points change and as such the performance of the score system might be lower than that of the original model. An alternative to this approach was recently proposed by Van Belle et al [30]. In this methodological paper, a color coding was used to visualize score systems. This representation of a score system is similar to the visualization of absolute risks by means of checkerboard plots in the WHO/ISH risk prediction charts [31]. Other model representations include the rank-hazard plot that visualizes the relative importance of predictors in a Cox regression model [32], the use of 3D and contour plots for the representation of interaction effects [33] and the visualization of individualized risk predictions by means of bar-line charts [34].

In this work, we propose to use color charts to visualize the prediction model and bar charts to visualize the risk prediction process for individual patients. These methods provide attractive tools to understand and communicate the relative influence of each predictor in general as well as for individual patients.

## Illustrative Examples from the Literature: The Framingham Heart Study

The different visualization methods will be illustrated on models from the Framingham heart study [35] to predict intermittent claudication [36] and stroke after atrial fibrillation [37]. The model representations used in this Section are the table-based representations as reported in the original articles [36,37] and on the website [35].

### Intermittent claudication

The Framingham Heart Study has developed a pooled logistic regression model to estimate the risk to develop intermittent claudication within 4 years, based on age (years), sex, serum cholesterol level (mg/dL), the hypertension level (normal, high normal, stage 1 or stage 2), the number of cigarettes smoked daily, whether the patient has diabetes and whether the patient has coronary heart disease (CHD) [36]. The coefficients of the logistic regression model are summarized in Table 1. To use this model on a patient, the predictor values need to be multiplied with the *β*-values given in the table to yield the contribution of each predictor (i.e. *β* ^*p*^ *x* ^*p*^, with *x* ^*p*^ the *p* ^th^ predictor). All these contributions are added to the intercept *β*_0_ to obtain the linear predictor, defined as

**Table 1:** Logistic regression coefficients for the intermittent claudication model.

Predictor		*β*
Intercept		-8.9152
Male sex		0.5033
Age		0.0372
Blood Pressure		
	*Normal*	0.0000
	*High Normal*	0.2621
	*Stage 1 Hypertension*	0.4067
	*Stage 2+ Hypertension*	0.7977
Diabetes		0.9503
Cigarettes/d		0.0314
Cholesterol, mg/dL		0.0048
CHD		0.9939

$$z=\beta_{0}+\sum_{p=1}^{d}\beta^{p}x^{p},$$ (1)

with *d* being the number of predictors. The estimated risk is then found as 1/(1+exp(-*z*)).

In order to facilitate the practical use of the model, the authors developed a score system. The resulting score model is summarized in Tables 2 and 3. To use on a patient, the points corresponding to the value of each predictor are summed up to obtain the score (Table 2). The risk is a monotonic function of this score and can be found in Table 3.

**Table 2:** Table based representation of the intermittent claudication score system. The points corresponding to the values of the predictors need to be added to each other to obtain the score (total number of points).

Predictor	range or level	points
Sex	male	3
	female	0
Age	45–49	0
	50–54	1
	55–59	2
	60–64	3
	65–69	4
	70–74	5
	75–79	6
	80–84	7
Cholesterol, mg/dL	< 170	0
	170–209	1
	210–249	2
	250–289	3
	> 289	4
Blood pressure	normal	0
	high normal	1
	stage 1	2
	stage 2+	4
Cigarettes/d, n	0	0
	1–5	1
	6–10	2
	11–20	3
	> 20	4
Diabetes	no	0
	yes	5
Coronary heart disease	no	0
	yes	5

**Table 3:** Conversion from points to risk for the intermittent claudication score system. To obtain a risk estimate, the obtained score is linked with a risk estimate.

score	estimate of 4-year probability
< 10	< 1%
10–12	1%
13–15	2%
16–17	3%
18	4%
19	5%
20	6%
21	7%
22	8%
23	10%
24	11%
25	13%
26	16%
27	18%
28	21%
29	24%
30	28%

### Stroke after atrial fibrillation

In this example, the Framingham heart study group used a Cox regression model to estimate the risk of having a stroke after atrial fibrillation [37]. The model is based on age (years), sex, systolic blood pressure (mmHg), whether the patient has diabetes mellitus and whether the patient has had a prior stroke or transient ischemic attack (TIA). The study population exists of 705 individuals between 55 and 94 years of age, who had an occurrence of new-onset atrial fibrillation, were not treated with warfarin at baseline, and did not have rheumatic mitral stenosis. The model coefficients are summarized in S1 Table. To estimate the predicted 5-year risk of stroke by means of the model, the predictor values are multiplied with their corresponding coefficient and summed. The survival probability is then estimated as

$$S(t)=S_{0}{\left(t\right)}^{\text{exp}\left(\sum_{p=1}^{d}\beta^{p}x^{p}-\sum_{p=1}^{d}\beta^{p}{\bar{x}}^{p}\right)}$$ (2)

with *S*_0_(*t*) being the estimated baseline survival function and ${\bar{x}}^{p}$ the average of *x* ^*p*^. For this example, the baseline survival at 5 years of follow-up was *S*_0_(5) = 0.8571 and the mean predictor values for sex, age, systolic blood pressure, diabetes and prior stroke were 1.48, 75, 146, 0.15 and 0.14, respectively. The score system derived from this model is presented in S2 and S3 Tables. Risk estimates can be obtained from S3 Table in the same way as from Table 3.

## Visualization Methods

### Model representation

**Fig 1:**
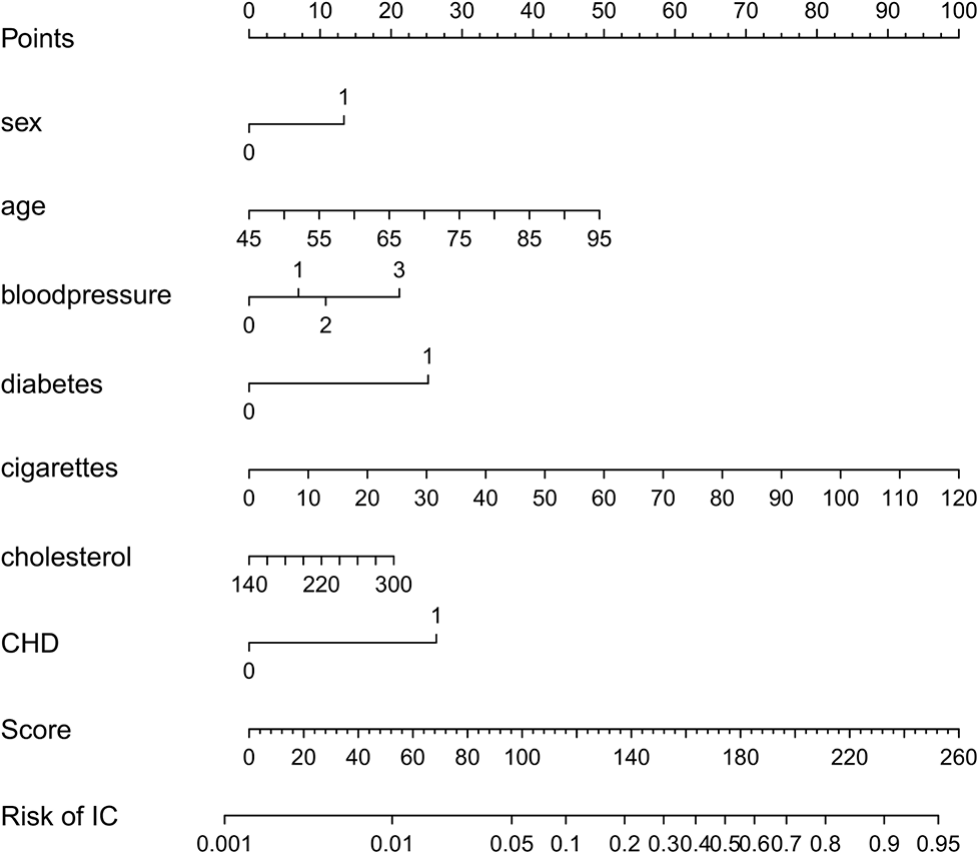
Nomogram for the intermittent claudication (IC) model. Each predictor is provided with a ruler. In order to obtain the contribution of a predictor to the prognostic index, the value of the predictor needs to be indicated and a vertical line needs to be drawn from this value, up to the points ruler. Addition of the points obtained for each predictor yields the score. The corresponding risk is found by drawing a vertical line from the score to the risk ruler.

Fig 1 illustrates the use of a nomogram to represent the intermittent claudication model. This figure was produced with the rms package in R (version 3.1.2). A nomogram consists of rulers for each predictor, a points ruler and two rulers to convert the score (i.e. the points total) to a risk estimate. Predictors with longer rulers have a higher impact on the risk estimate since the range of the contributions of this predictor is broader. For each predictor the contribution to the linear predictor can be found by drawing a vertical line from the predictor value up to the points ruler at the top of the graph. Note that the obtained points are a scaled version of

$$\beta^{p}x^{p}-\underset{i\in D}{\text{min}}\left(\beta^{p}x_{i}^{p}\right),$$ (3)

where the second term in Eq 3 indicates the minimal contribution of predictor *x* ^*p*^ observed in the data. The sum of all these points yields the ‘score’. The estimated risk of intermittent claudication is found by drawing a line from the ‘score’ ruler to the risk ruler.

**Fig 2:**
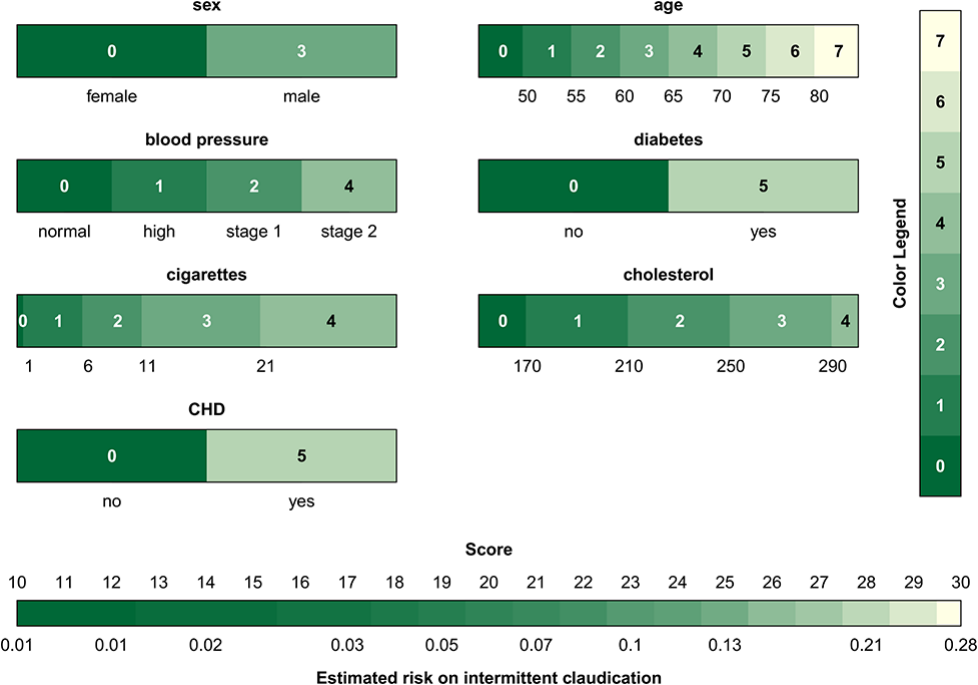
Graphical representation of the intermittent claudication score system. The colors relate to the points that are attributed to a predictor value. Dark green indicates a low contribution and light green to ecru a high contribution to the risk. The points corresponding to the colors are indicated within each interval and by means of the color legend. It is instantly seen that older age and having diabetes or coronary heart disease have the highest contribution to the risk of intermittent claudication. Addition of all points corresponding to the predictor values of a patient gives the score which can then be converted into a risk estimate by means of the color bar at the bottom.

Although we acknowledge the advantages of a nomogram to understand the relative contributions of the predictors, we argue that the addition of color codes will improve direct interpretation. Interpreting colors is very intuitive since we are used to do so in daily life (e.g. traffic lights). Research in different areas also demonstrates the effect of colors on marketing, brand recognition, attention, learning and comprehension. It is also reported that visual displays can enhance the communication of risk [38,39]. We therefore propose to use the color-based representation, as used in a paper by Van Belle et al for score systems [30]. Instead of a table-based approach, Fig 2 uses the color-based method to present the score system for intermittent claudication. The color corresponding to each predictor value corresponds to a number of points, which is indicated within each colored interval. The patient's score is the sum of the points for all predictors. This score is converted into a risk estimate by means of the last color bar where a dark green color indicates a low risk and a light green to ecru color indicates high risk. This visualization of the model allows users to instantly gauge which predictors contribute most to a high risk estimate. In this case, older age and having diabetes or coronary heart disease strongly contribute to the risk of intermittent claudication.

**Fig 3:**
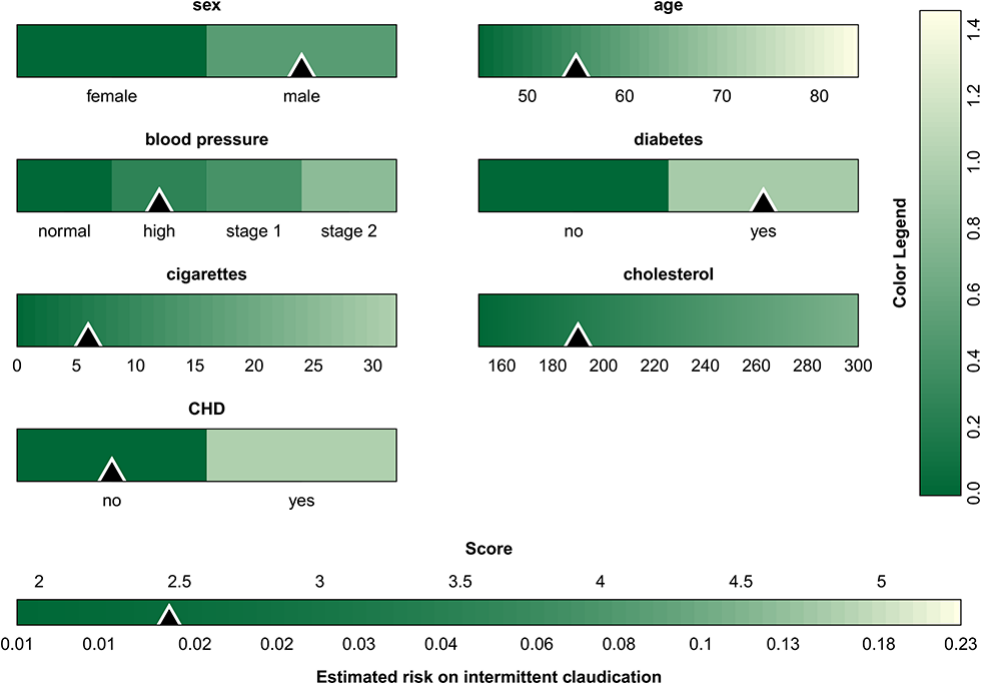
Graphical representation of the intermittent claudication model. For each predictor the range is indicated below the color bar, and the color indicates the contribution to the linear predictor corresponding to the predictor value. The colors are converted to points by means of the color legend at the right of the graph. The sum of all points, i.e. the score, is then converted to the estimated risk by means of the color bar at the bottom The triangles indicate the predictor values and the corresponding risk estimate for a 55 year old man with a high blood pressure and diabetes, who smokes 6 cigarettes a day and has a cholesterol value of 190 mg/dL.

Given the advantages of the color-based representation of a score system, we propose to extend this approach to continuous model representations. In addition to a table with model coefficients (Table 1), the model can be visualized as in Fig 3. At the bottom of each bar the range of the predictor is indicated. The colors encode the contribution of the predictors to the linear predictor using Eq 3, and the conversion from the color to the points is made by means of the color bar at the right of the graph. The figure is used in the same way as Fig 2, and visualizes how the various predictors influence the risk of intermittent claudication. Similar to nomograms, it is difficult to extract the exact number of points for each predictor when representing continuous models. The bars mainly help to visualize the operation of the model, and to assess the relative influence of the different predictor values. When implementing the model online, it would be useful to add boxes next to each bar where the predictor values can be entered. As a result, the entered predictor values can be indicated on the graph such that the most influential values are instantly identified. This approach is visualized by means of the triangles in Fig 3, which indicate the measurements for a fictitious patient (a 55-year old man, with a high normal blood pressure and diabetes, who smokes 6 cigarettes a day and has a cholesterol value of 190 mg/dL). Additionally, percentiles of the predictor variables as observed in the training set can be added to the model visualization in order to identify extreme values. This is visualized in S1 Fig for the stroke model by means of the dashed gray vertical lines. The percentiles as shown on the figure are merely exemplary since the original data were not available to the authors. Finally, S2 Fig illustrates the color-based representation of the score system for the risk of stroke after atrial fibrillation.

In the proposed visualization technique, it was chosen to represent the lowest contribution of each predictor to the linear predictor by zero points. This is in analogy with nomograms. Alternatively, zero points can be addressed to the predictor means or some other relevant value. The definition of the zero value is important to correctly interpret the resulting visualization.

In Figs 2 and 3 and S1 and S2 Figs, a sequential color map is used, the color of which is based on recommendations of [40] w.r.t. whether the map is color-blind friendly, LCD friendly and print friendly. Since the color map is to be used to indicate the change in contribution, a rainbow color map, which may be familiar to most end users, was discarded since apparent sharp changes in the visualization occur due to rainbow color map artifacts [41]. Additionally, the rainbow color map lacks perceptual ordering such that users have to refer to the color legend more often to interpret the result. In case one would like to set one particular patient (e.g. the mean of the observed values in the training data) as a reference, one could opt to set the contributions of these predictor values to zero. The use of a diverging color map (a color map with a well-defined midpoint) in this particular case can aid to identify which contributions of a new patient are below or above the reference. Although these features are implemented within the software, we will not elaborate on these options here.

### Patient-specific contribution charts

**Fig 4:**
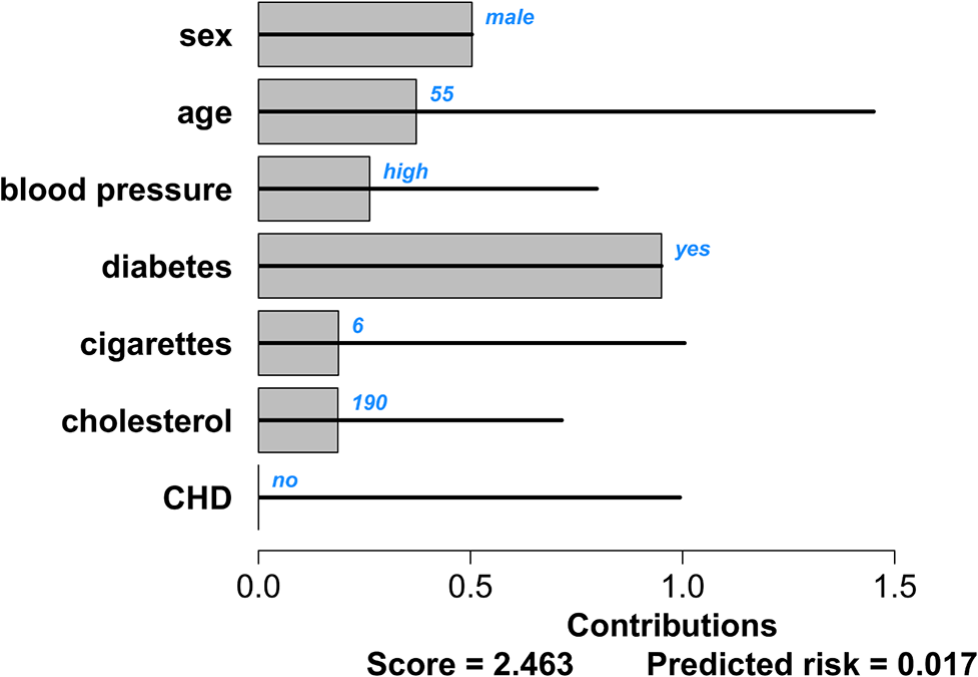
Contribution chart for the intermittent claudication model for a patient with a good prognosis: a 55 year old man with a high blood pressure and diabetes, who smokes 6 cigarettes a day and has a cholesterol value of 190 mg/dL. The black lines indicate the range of contributions for each predictor as observed in the data set. The bars indicate the predictors’ contributions to the linear predictor for this specific patient. The patient-specific predictor values are indicated in blue. The score at the bottom of the graph is the sum of all predictor contributions. The estimated risk of intermittent claudication corresponding to this score is given as well.

**Fig 5:**
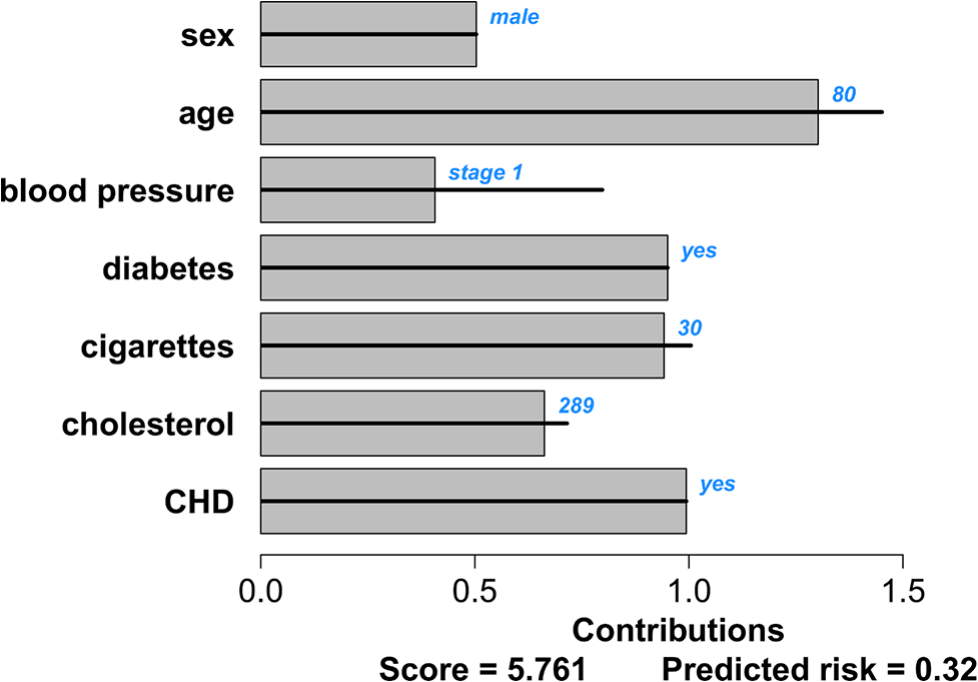
Contribution chart for the intermittent claudication model for a patient with a bad prognosis: an 80-year old man with stage 1 hypertension, diabetes, a cholesterol value of 289 mg/dL and coronary heart disease, who smokes 30 cigarettes a day. The black lines indicate the range of contributions for each predictor as observed in the data set. The bars indicate the predictors’ contributions to the linear predictor for this specific patient. The patient-specific predictor values are indicated in blue. The score at the bottom of the graph is the sum of all predictor contributions. The estimated risk of intermittent claudication corresponding to this score is given as well.

Specifying how the estimated risk is obtained for specific patients can be important to decide upon the most appropriate treatment for each individual [34]. We use the additive model structure, where the linear predictor is found as a weighted sum of predictor values, as the basis of the representation schemes that we propose. The contribution of each predictor *x* ^*p*^ to this linear predictor is then *β* ^*p*^ *x* ^*p*^. We present two graphical representations of patient-specific contributions. The first representation is based on the visualization of marginal effects for black-box models by Strumbelj and Kononenko [42]. In order to visualize which predictors contribute the most to the score for a particular patient, the contributions can be represented by means of bar charts. The largest bar corresponds to the predictor that contributes the most to the score and hence to the risk. This approach is visualized for the intermittent claudication model in Figs 4 and 5 for two different patients: a 55-year old man, with a high normal blood pressure and diabetes, who smokes 6 cigarettes a day and has a cholesterol value of 190 mg/dL; and an 80-year old man with stage 1 hypertension, diabetes, a cholesterol value of 289 mg/dL and coronary heart disease, who smokes 30 cigarettes a day. The observed values are indicated in blue at the right-hand side next to the predictor bar. The gray bars indicate the contributions of each predictor, which are computed as in Eq 3. At the bottom of the figure, the sum of all these contributions (the score) and the corresponding risk estimate are given. Note that for models including an intercept, it is not necessary to include this in the graph as it is taken into account in the conversion from score to risk estimate. The horizontal black lines through each bar indicate the minimal and maximal contribution of this predictor as observed in the training set. From these visualizations it is clearly seen that the second patient has more predictor values that contribute to a high risk estimate.

**Fig 6:**
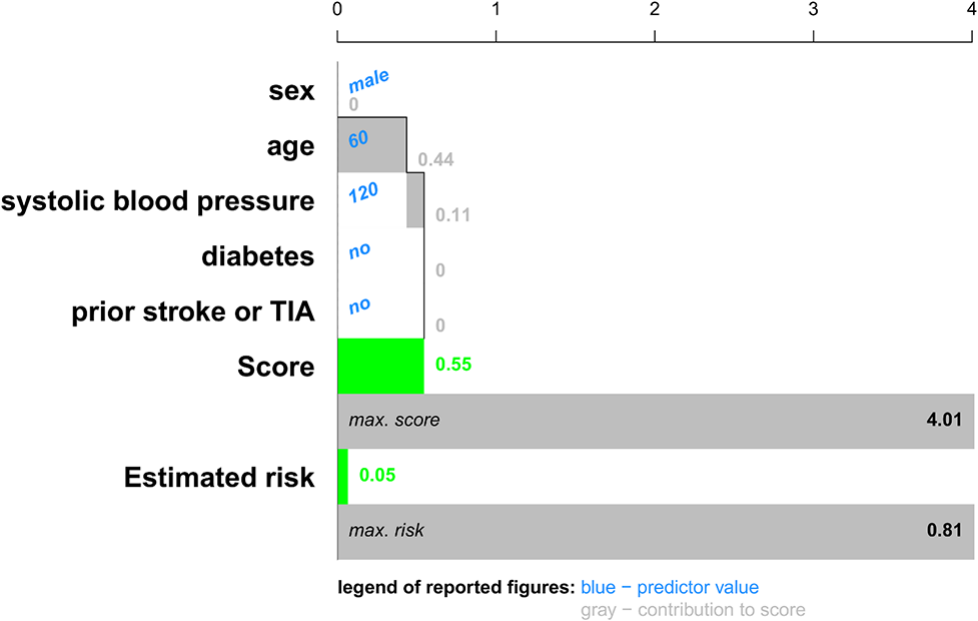
Cumulative contribution chart representing patient-specific predictor contributions for the stroke model for a patient with a good prognosis: a 60-year old man with a systolic blood pressure of 120 mm Hg. For each predictor the contribution to the linear predictor or score is represented by means of a bar, each of which starts where the previous ended. The score is the sum of all these contributions. An extra bar indicates the maximal score observed in the data set. The estimated risks corresponding to this patient's score and the most extreme score observed in the data set is visualized as well. For this application, we chose to represent an estimated risk of 10% or higher by a red color, representing a bad prognosis. Lower risk estimates are visualized in green.

**Fig 7:**
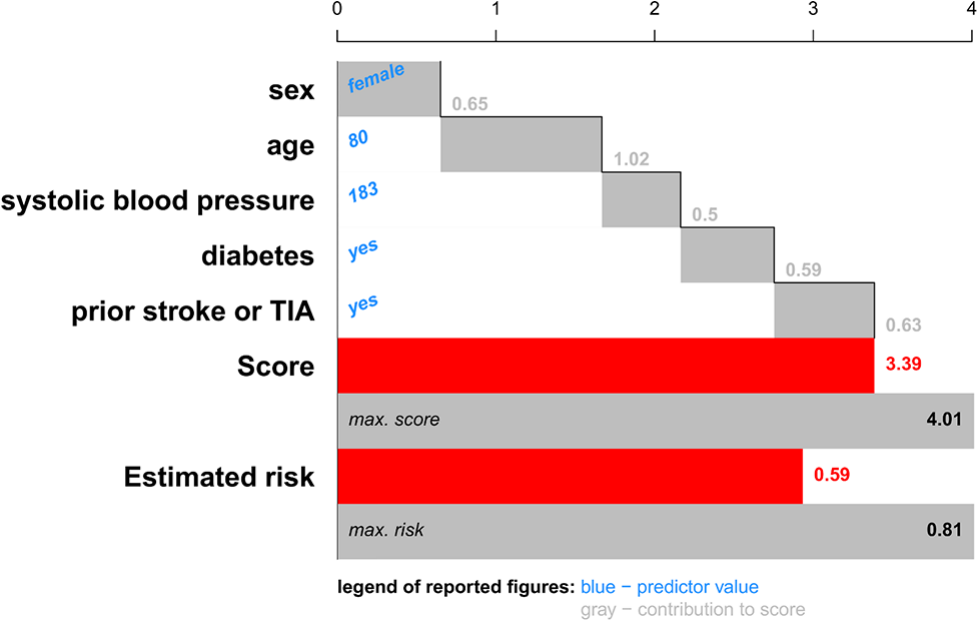
Cumulative contribution chart representing patient-specific predictor contributions for the stroke model for a patient with a bad prognosis: an 80-year old woman with a systolic blood pressure of 183 mm Hg, diabetes and a prior stroke or TIA. For each predictor the contribution to the linear predictor or score is represented by means of a bar, each of which starts where the previous ended. The score is the sum of all these contributions. An extra bar indicates the maximal score observed in the data set. The estimated risks corresponding to this patient's score and the most extreme score observed in the data set is visualized as well. For this application, we chose to represent an estimated risk of 10% or higher by a red color, representing a bad prognosis. Lower risk estimates are visualized in green.

The second representation of patient-specific predictor contributions uses the cumulative score values to visualize the addition of different contributions to the final score. This approach is used for the risk of stroke after atrial fibrillation in Figs 6 and 7 for two different patients: a 60-year old man with a systolic blood pressure of 120 mm Hg; and an 80-year old woman with a systolic blood pressure of 183 mm Hg, diabetes and a prior stroke or TIA. Typical for this cumulative chart is that each bar starts where the previous bar ended. This plot shows how the score is built up from the predictor contributions. Due to the cumulative nature, the score is naturally obtained after addition of the contributions of the last predictor. The contributions are computed as in Eq 3. For each predictor, the value is indicated in blue to the right of the predictor’s name. The contribution to the score is indicated in grey. The score and the corresponding risk estimates are shown by colored bars below the predictor contributions. Additional bars indicate the highest score and risk estimate observed in the training set. In this example, it was chosen to show a risk below 10% in green. Higher risk estimates are indicated in red. In practice, more colors can be used, which might indicate different procedures to be followed for patients with different risk profiles.

In some cases, it might be insightful to sort the predictors by increasing contributions to the score. This option is illustrated for the contribution chart in S3 Fig and for the cumulative contributions chart in S4 Fig.

## Extensions towards Nonlinear Models and Interaction Effects

**Fig 8:**
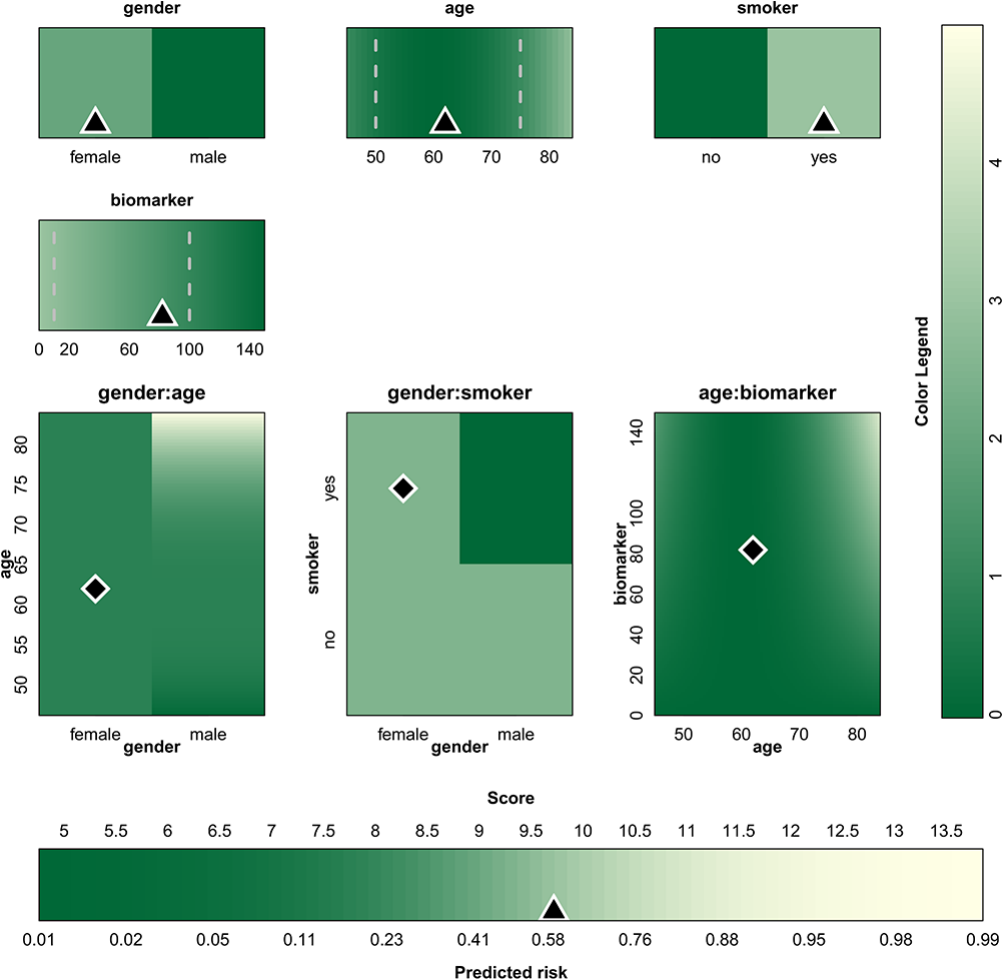
Graphical representation of an artificial logistic model that includes non-linear functional forms and interaction effects. For each predictor or interaction, the range is indicated below or next to the color bars and color plots. The color indicates the contribution to the linear predictor corresponding to the predictor values. The colors are converted to points by means of the color legend at the right of the graph. The sum of all points, i.e. the score, is then converted to the estimated risk by means of the color bar at the bottom. The triangles/diamonds indicate the predictor values and the corresponding risk estimate for a specific patient. The dashed gray lines are used to show percentiles.

For non-linear models the predictor contributions become *β* ^*p*^ *f* ^*p*^(*x* ^*p*^), with *f* ^*p*^(*x* ^*p*^) a non-linear transformation of the predictor *x* ^*p*^, such as logarithmic or power transformations or restricted cubic splines. A non-linear effect can be represented in the same way as before, where the color of each color bar will now represent *β* ^*p*^ *f* ^*p*^(*x* ^*p*^) instead of *β* ^*p*^ *x* ^*p*^. The presented visualizations can be further extended to include interaction effects. For each interaction effect, an additional color plot is added to the model representation. For the patient-specific representations, a bar is added with length

$$\beta^{p,q}f^{p,q}\left(x^{p},x^{q}\right)-\underset{i\in D}{\text{min}}\left(\beta^{p,q}f^{p,q}\left(x_{i}^{p},x_{i}^{q}\right)\right),$$ (4)

with *β* ^*p*,*q*^ the coefficient of the interaction and *f* ^*p*,*q*^(*x* ^*p*^, *x* ^*q*^) a transformation on both predictors involved in the interaction. This approach is illustrated in Fig 8 for an artificial example with 4 predictors: gender (binary), age (continuous), smoker (binary) and a biomarker (continuous). The visualized model is a logistic regression model with *z* = -1 + *β*_1_ *gender + *β*_2_ *(*age*– 60)^2^ + *β*_3_ *smoker + *β*_4_ *biomarker + *β*_5_ *gender*((*age*-60)/10)^3^ + *β*_6_ *gender*smoker + *β*_7 *_(*age*-60)^2*^ *biomarker*, with *β*_1_ = -2, *β*_2_ = 0.005, *β*_3_ = 3, *β*_4_ = -0.02, *β*_5_ = 0.3, *β*_6_ = -2.5, and *β*_7_ = 0.00005. In addition to linear main effects, this model contains a non-linear main effect for age, an interaction effect between gender and age, an interaction effect between gender and smoker and an interaction effect between age and biomarker. In addition to the model visualization, the 5^th^ and 95^th^ percentile (for continuous predictors) have been added in dashed gray lines in Fig 8. The predictor values for one specific patient (a 62 year old smoking woman with a biomarker level of 82 U/mL) together with the estimated risk are indicated by means of the triangles and diamonds. The contribution chart for this patient is given in S5 Fig, where a contribution for all relevant interaction effects is added in addition to the contributions of the main effects.

The methods can even be further extended towards kernel-based methods such as support vector machines [43,44], as long as the chosen kernel is additive.

## Software

The figures in this work are generated in R (version 3.1.2). An R package gathering all functionality described above is available at our website (http://homes.esat.kuleuven.be/~sistawww/biomed/iota/index.php/mr/14-uncategorised/mr/61-visualization). The package also provides different options regarding the color map and the level of the contributions that should be represented as zero. More information can be found in the manual that can be downloaded from the same website.

## Discussion

Interpretation of risk prediction models, even the simplest ones, is not straightforward for clinicians and their patients. The most common representation type is a table of odds/hazard ratios that are not intuitive and often interpreted incorrectly. Research shows that patients prefer graphical representations of risk above numerical risk estimates, as this improves their comprehension of risk [38]. This work presents visualization methods to represent risk prediction models and patient-specific contribution charts to enhance interpretation of these models. The color-based model representation offers an alternative to nomograms for visualizing prediction models. Nomograms use line segments of varying length to show the influence of the predictors, whereas the color plots use bars of fixed length with varying color schemes. Colors are known to be attractive and understandable, and are used often in risk charts [45]. The color plots may be more convenient in communicating nonlinear effects such as quadratic terms and interactions between two continuous predictors. The approach to visualize interaction terms bears resemblance to recent work by Lamina and colleagues, and is easily incorporated in the color plots [33].

The color schemes in the plots visualize how the model obtains a risk estimate, and thus allows indicating the most contributing predictors for a specific patient. When interpreting colors, care needs to be taken with respect to the reference values of the predictors. The interpretation depends on which value of the functional form is taken to be zero. Although the underlying model is the same, contributions can be negative as well as positive in case a reference (e.g. the median observation) is set, making it more difficult to see which predictors contribute the most. The indication of percentiles and predictor values of one particular patient in an online implementation tool enables to indicate extreme values and to interpret how a patient’s estimated risk is established, respectively.

To further enhance interpretation and communication, we also proposed to visualize the risk estimation process for a specific patient by means of bar charts. The first type of contribution charts represents the contributions directly and offer the advantage that the maximal contribution within the training set is represented, such that extreme values can be detected easily. The cumulative contribution charts directly show how the linear predictor is built based on the predictors and how the risk is situated within the risk estimates observed in the training set. Additional colors can be used to give advice on the optimal treatment for a specific patient. For this purpose, communication with clinical experts is a necessity but this communication is also facilitated by these graphs. The patient-specific plots relate to the bar-line charts from Björk and colleagues [34]. Their bar-line plots, however, are based on a sequential inclusion of predictors and hence results are order-dependent. This is inconsistent with nomograms and color plots, which show independent contributions of all predictors to the risk. The sequential approach restricts their use to communicate individual contributions, despite the fact that a logical order could be discerned with respect to the priority of types of predictors: depending on context, it can be defended that the value of simple demographic predictors is investigated first, followed by specific disease characteristics and finally by advanced predictors such as genetic parameters or complex imaging-based measurements.

Although the literature states that graphically representing risks enhances the comprehension of risk [38], evidence of the improved interpretability and acceptance of risk prediction models by clinicians by visualizing them as proposed here can only be provided after conducting a user study, an example of which can be found in [46]. Different application areas could prefer different settings and might as well prefer different visualization methods. User studies should therefore be application specific and investigate whether the used visualization technique and applied color map are able to report what the end user needs. Questions that need to be answered are: do the users understand this representation and do they want to adopt this representation in their work flow.

Using visualization methods has several advantages over current methods such as odds and hazard ratios to summarize risk prediction methods. Without model visualization, the risk estimate may be the only directly interpretable result of a risk prediction model available to the clinician. However, optimal treatment might be very different for patients with the same risk estimate. A simple example involves the treatment of breast cancer. Two patients with the same risk estimate may require different treatment, for example because of a different estrogen receptor status. Hormonal therapy will only benefit the patient with estrogen-positive cancer. We realize that prediction models only relate measurements to a disease of interest without necessarily implying a direct causal relationship. Nevertheless, understanding the operation of prediction models is essential for thoughtful and successful use in practice. To this end, the proposed visualization techniques are appealing tools in the communication between model developers, model implementers and the clinical end-user, and will therefore result in software tools that better fit the clinical workflow. Additionally, the clinician can use these techniques to enhance communication with patients and to explain why a certain treatment is advised.

## Supporting Information

S1 FigGraphical representation of the stroke model.For each predictor, the colors indicate the contribution to the prognostic index, i.e. $\beta^{p}x^{p}-\underset{i\in D}{\text{min}}(\beta^{p}x_{i}^{p})$, where $\underset{i\in D}{\text{min}}(\beta^{p}x_{{}^{i}}^{p})$ indicates the minimal contribution of predictor *x* ^*p*^ observed in the data. The points associated with these colors can be extracted by means of the color legend at the right of the graph. The score, obtained by summing all points, is translated into the risk estimate by means of the color bar at the bottom of the graph. The dashed gray lines indicate how percentiles can be visualized to detect extreme values in new patients. Note that the exact values for the percentiles are not representative for this dataset since the original data were not available to the authors.(PDF)

S2 Fig Graphical representation of the stroke score system. The points corresponding to each color are indicated within each interval and in the color legend. Summation of all points results in the score, which is then converted into a risk estimate by means of the bottom most color bar.
(PDF)

S3 Fig Contribution chart for the intermittent claudication model. The predictors are sorted according to increasing contributions to the score. The black lines indicate the range of contributions for each predictor as observed in the data set. The bars indicate the predictors’ contributions to the linear predictor for this specific patient. The patient-specific predictor values are indicated in blue. The score at the bottom of the graph is the sum of all predictor contributions. The estimated risk of intermittent claudication corresponding to this score is given as well. (a) A 55 year old man with a high blood pressure and diabetes, who smokes 6 cigarettes a day and has a cholesterol value of 190 mg/dL. (b) An 80-year old man with stage 1 hypertension, diabetes, a cholesterol value of 289 mg/dL and coronary heart disease, who smokes 30 cigarettes a day.
(PDF)

S4 Fig Cumulative contribution chart representing patient-specific predictor contributions for the stroke model. The predictors are sorted according to increasing contributions to the score. For each predictor the contribution to the linear predictor or score is represented by means of a bar, each of which starts where the previous ended. The score is the sum of all these contributions. An extra bar indicates the maximal score observed in the data set. The estimated risks corresponding to this patient's score and the most extreme score observed in the data set is visualized as well. For this application, we chose to represent an estimated risk of 10% or higher by a red color, representing a bad prognosis. Lower risk estimates are visualized in green. (a) A 60-year old man with a systolic blood pressure of 120 mm Hg. (b) An 80-year old woman with a systolic blood pressure of 183 mm Hg, diabetes and a prior stroke or TIA.
(PDF)

S5 Fig Contribution chart for the artificial model for a 62-year old smoking female with a biomarker level of 82. The black lines indicate the range of contributions for each predictor as observed in the data set. The bars indicate the predictors’ contributions to the linear predictor for this specific patient. The patient-specific predictor values are indicated in blue. The score at the bottom of the graph is the sum of all predictor contributions. The estimated risk corresponding to this score is given as well.
(PDF)

S1 Table Coefficients of the Cox model for the prediction of stroke after atrial fibrillation. TIA: transient ischemic attack.
(PDF)

S2 Table Table based representation of the stroke after atrial fibrillation score system. The points corresponding to the values of the predictors need to be added to each other to obtain the score (total number of points).
(PDF)

S3 Table Conversion from points to risk for the stroke after atrial fibrillation score system. To obtain a risk estimate, the obtained score is linked with a risk estimate.
(PDF)
