# Supplementary material for: Visualizing Risk Prediction Models
Source: PLoS One. 2015 Jul 15;10(7):e0132614. doi: 10.1371/journal.pone.0132614 (PMC4503430; doi:10.1371/journal.pone.0132614)
Supplement: S1 Fig — For each predictor, the colors indicate the contribution to the prognostic index, i.e. βpxp−mini∈D(βpxip), where mini∈D(βpxip) indicates the minimal contribution of predictor x p observed in the data. The points associated with these colors can be extracted by means of the color legend at the right of the graph. The score, obtained by summing all points, is translated into the risk estimate by means of the color bar at the bottom of the graph. The dashed gray lines indicate how percentiles can be visualized to detect extreme values in new patients. Note that the exact values for the percentiles are not representative for this dataset since the original data were not available to the authors. (PDF) [file pone.0132614.s001.pdf]

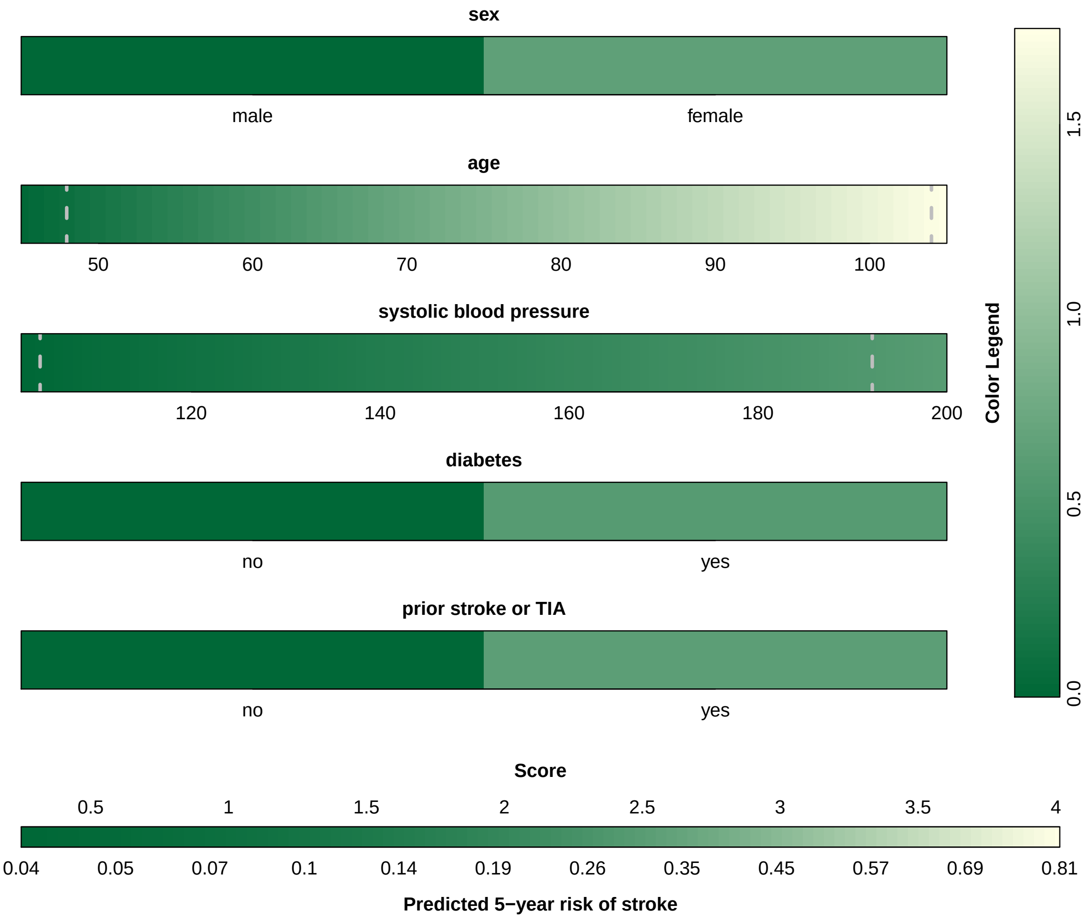

S1 Fig.: Graphical representation of the stroke model. For each predictor, the colors indicate the contribution to the prognostic index, i.e. $\beta^p x^p - \min_{i\in\mathcal{D}}(\beta^p x_i^p)$, where $\min_{i\in\mathcal{D}}(\beta^p x_i^p)$ indicates the minimal contribution of predictor x^p observed in the data. The points associated with these colors can be extracted by means of the color legend at the right of the graph. The score, obtained by summing all points, is translated into the risk estimate by means of the color bar at the bottom of the graph. The dashed gray lines indicate how percentiles can be visualized to detect extreme values in new patients. Note that the exact values for the percentiles are not representative for this dataset since the original data were not available to the authors.
