# Supplementary material for: Visualizing Risk Prediction Models
Source: PLoS One. 2015 Jul 15;10(7):e0132614. doi: 10.1371/journal.pone.0132614 (PMC4503430; doi:10.1371/journal.pone.0132614)
Supplement: S2 Fig — The points corresponding to each color are indicated within each interval and in the color legend. Summation of all points results in the score, which is then converted into a risk estimate by means of the bottom most color bar. (PDF) [file pone.0132614.s002.pdf]

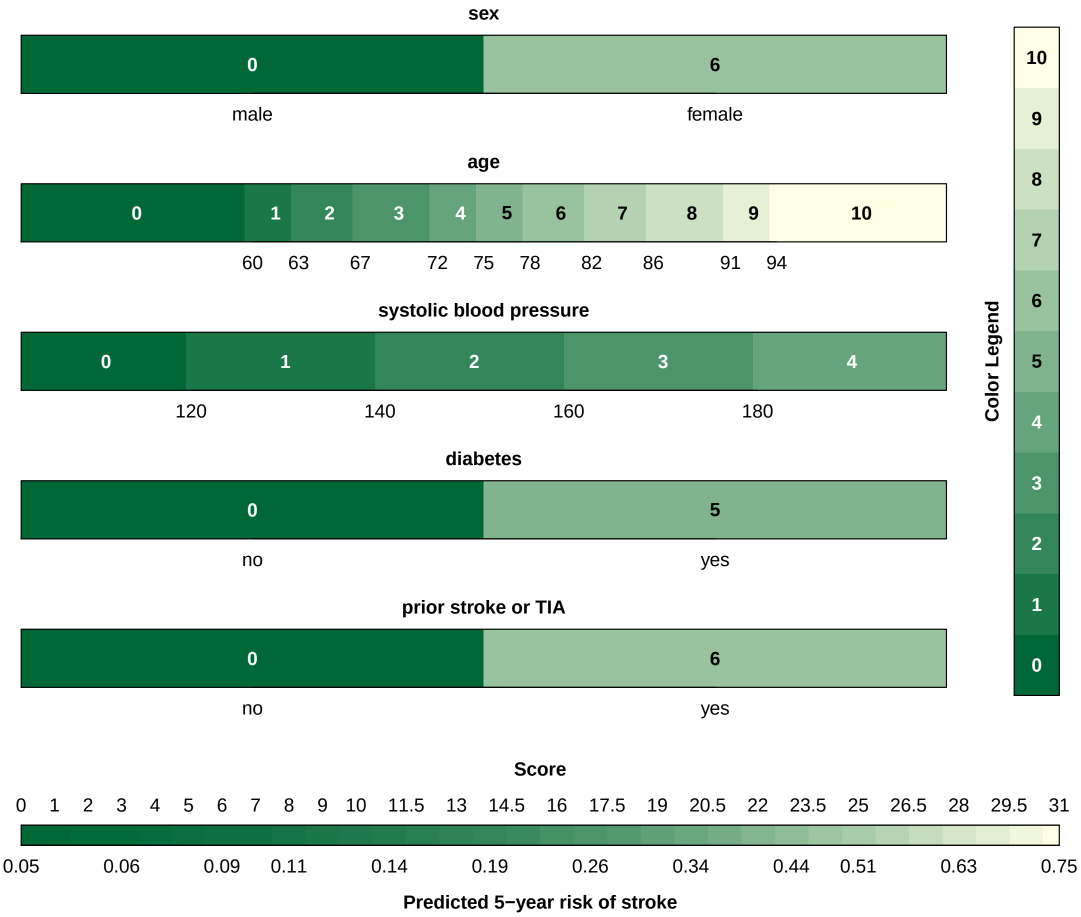

S2 Fig.: Graphical representation of the stroke score system. The points corresponding to each color are indicated within each interval and in the color legend. Summation of all points results in the score, which is then converted into a risk estimate by means of the bottom most color bar.
