# Supplementary material for: Visualizing Risk Prediction Models
Source: PLoS One. 2015 Jul 15;10(7):e0132614. doi: 10.1371/journal.pone.0132614 (PMC4503430; doi:10.1371/journal.pone.0132614)
Supplement: S3 Fig — The predictors are sorted according to increasing contributions to the score. The black lines indicate the range of contributions for each predictor as observed in the data set. The bars indicate the predictors’ contributions to the linear predictor for this specific patient. The patient-specific predictor values are indicated in blue. The score at the bottom of the graph is the sum of all predictor contributions. The estimated risk of intermittent claudication corresponding to this score is given as well. (a) A 55 year old man with a high blood pressure and diabetes, who smokes 6 cigarettes a day and has a cholesterol value of 190 mg/dL. (b) An 80-year old man with stage 1 hypertension, diabetes, a cholesterol value of 289 mg/dL and coronary heart disease, who smokes 30 cigarettes a day. (PDF) [file pone.0132614.s003.pdf]

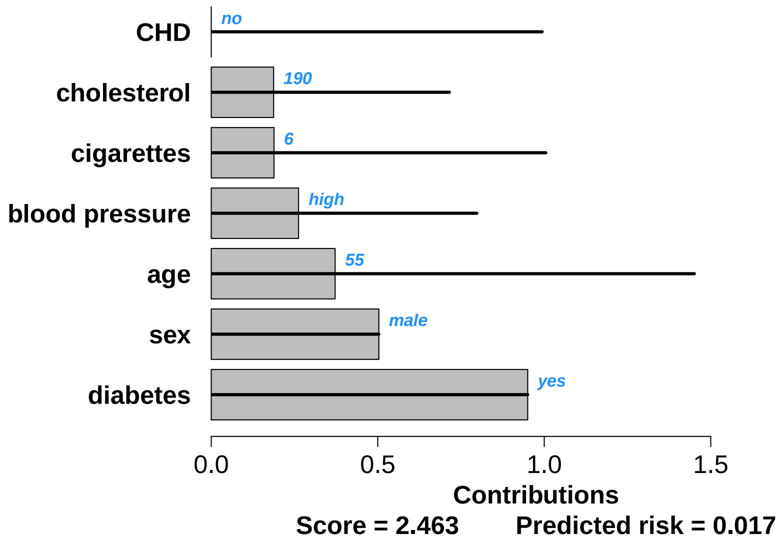

(a)

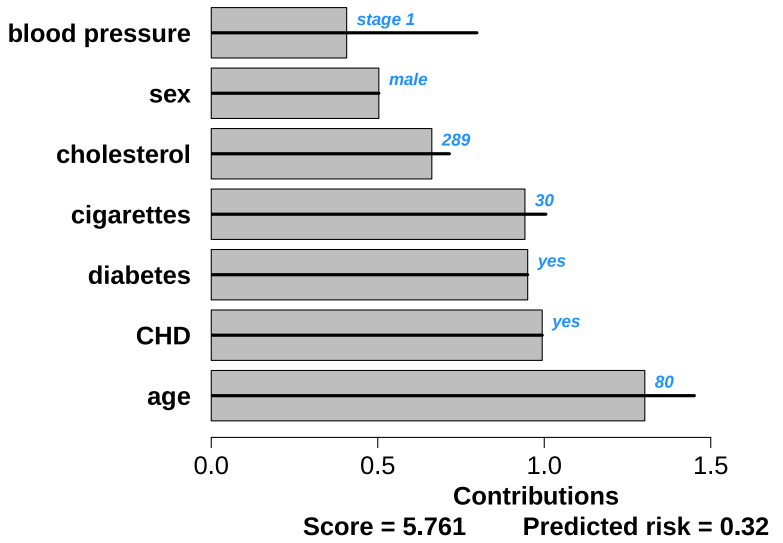

(b)

S3 Fig.: Contribution chart for the intermittent claudication model. The predictors are sorted according to increasing contributions to the score. The black lines indicate the range of contributions for each predictor as observed in the data set. The bars indicate the predictors's contributions to the linear predictor for this specific patient. The patient-specific predictor values are indicated in blue. The score at the bottom of the graph is the sum of all predictor contributions. The estimated risk of intermittent claudication corresponding to this score is given as well. (a) A 55 year old man with a high blood pressure and diabetes, who smokes 6 cigarettes a day and has a cholesterol value of 190 mg/dL. (b) An 80-year old man with stage 1 hypertension, diabetes, a cholesterol value of 289 mg/dL and coronary heart disease, who smokes 30 cigarettes a day.
