# Supplementary material for: Visualizing Risk Prediction Models
Source: PLoS One. 2015 Jul 15;10(7):e0132614. doi: 10.1371/journal.pone.0132614 (PMC4503430; doi:10.1371/journal.pone.0132614)
Supplement: S4 Fig — The predictors are sorted according to increasing contributions to the score. For each predictor the contribution to the linear predictor or score is represented by means of a bar, each of which starts where the previous ended. The score is the sum of all these contributions. An extra bar indicates the maximal score observed in the data set. The estimated risks corresponding to this patient's score and the most extreme score observed in the data set is visualized as well. For this application, we chose to represent an estimated risk of 10% or higher by a red color, representing a bad prognosis. Lower risk estimates are visualized in green. (a) A 60-year old man with a systolic blood pressure of 120 mm Hg. (b) An 80-year old woman with a systolic blood pressure of 183 mm Hg, diabetes and a prior stroke or TIA. (PDF) [file pone.0132614.s004.pdf]

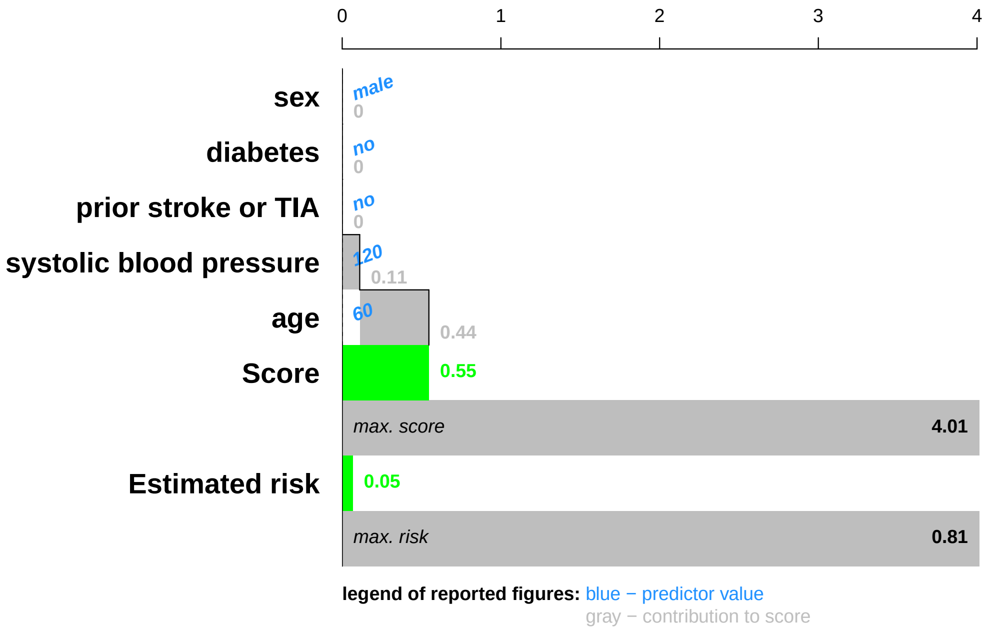

(a)

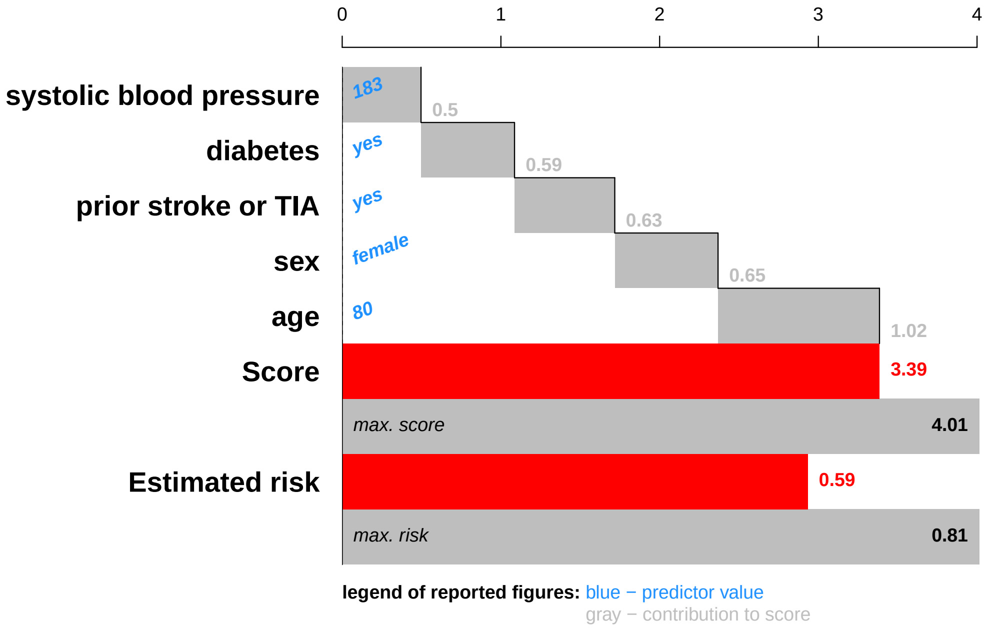

(b)

S4 Fig.: Cumulative contribution chart representing patient-specific predictor contributions for the stroke model. The predictors are sorted according to increasing contributions to the score. For each predictor the contribution to the linear predictor or score is represented by means of a bar, each of which starts where the previous ended. The score is the sum of all these contributions. An extra bar indicates the maximal score observed in the data set. The estimated risks corresponding to this patient's score and the most extreme score observed in the data set is visualized as well. For this application, we chose to represent an estimated risk of 10% or higher by a red color, representing a bad prognosis. Lower risk estimates are visualized in green. (a) A 60-year old man with a systolic blood pressure of 120 mm Hg. (b) An 80-year old woman with a systolic blood pressure of 183 mm Hg, diabetes and a prior stroke or TIA.
