# Supplementary material for: Visualizing Risk Prediction Models
Source: PLoS One. 2015 Jul 15;10(7):e0132614. doi: 10.1371/journal.pone.0132614 (PMC4503430; doi:10.1371/journal.pone.0132614)
Supplement: S5 Fig — The black lines indicate the range of contributions for each predictor as observed in the data set. The bars indicate the predictors’ contributions to the linear predictor for this specific patient. The patient-specific predictor values are indicated in blue. The score at the bottom of the graph is the sum of all predictor contributions. The estimated risk corresponding to this score is given as well. (PDF) [file pone.0132614.s005.pdf]

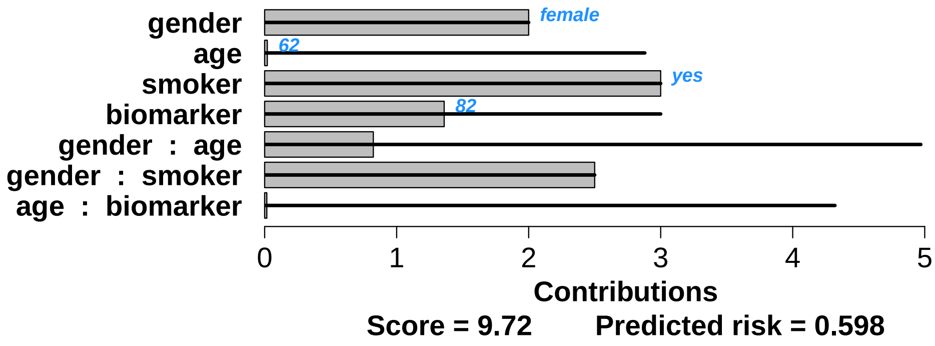

S5 Fig.: Contribution chart for the artificial model for a 62-year old smoking female with a biomarker level of 82. The black lines indicate the range of contributions for each predictor as observed in the data set. The bars indicate the predictors's contributions to the linear predictor for this specific patient. The patient-specific predictor values are indicated in blue. The score at the bottom of the graph is the sum of all predictor contributions. The estimated risk corresponding to this score is given as well.
