# Supplementary material for: Visualizing Risk Prediction Models
Source: PLoS One. 2015 Jul 15;10(7):e0132614. doi: 10.1371/journal.pone.0132614 (PMC4503430; doi:10.1371/journal.pone.0132614)
Supplement: S1 Table — TIA: transient ischemic attack. (PDF) [file pone.0132614.s006.pdf]

S1 Table: Coefficients of the Cox model for the prediction of stroke after atrial fibrillation. TIA: transient ischemic attack.

Predictor	β
Sex (1=Men, 2=Women)	0.6507
Age, per y	0.0291
Systolic Blood Pressure, per mmHg	0.0061
Diabetes Mellitus	0.5893
Prior Stroke/TIA	0.6316
