# Supplementary material for: Visualizing Risk Prediction Models
Source: PLoS One. 2015 Jul 15;10(7):e0132614. doi: 10.1371/journal.pone.0132614 (PMC4503430; doi:10.1371/journal.pone.0132614)
Supplement: S2 Table — The points corresponding to the values of the predictors need to be added to each other to obtain the score (total number of points). (PDF) [file pone.0132614.s007.pdf]

S2 Table: Table based representation of the stroke after atrial fibrillation score system. The points corresponding to the values of the predictors need to be added to each other to obtain the score (total number of points).

Predictor	range or level	points
Age	55-59	0
	60-62	1
	63-66	2
	67-71	3
	72-74	4
	75-77	5
	78-81	6
	82-85	7
	86-90	8
	91-93	9
	> 93	10
Sex	Men	0
	Women	6
Systolic Blood Pressure, mm Hg	< 120	0
	120-139	1
	140-159	2
	160-179	3
	> 179	4
Diabetes	No	0
	Yes	5
Prior Stroke or TIA	No	0
	Yes	6
