# Supplementary material for: Visualizing Risk Prediction Models
Source: PLoS One. 2015 Jul 15;10(7):e0132614. doi: 10.1371/journal.pone.0132614 (PMC4503430; doi:10.1371/journal.pone.0132614)
Supplement: S3 Table — To obtain a risk estimate, the obtained score is linked with a risk estimate. (PDF) [file pone.0132614.s008.pdf]

S3 Table: Conversion from points to risk for the stroke after atrial fibrillation score system. To obtain a risk estimate, the obtained score is linked with a risk estimate.

score	estimated 5-Year risk
0-1	5%
2-3	6%
4	7%
5	8%
6-7	9%
8	11%
9	12%
10	13%
11	14%
12	16%
13	18%
14	19%
15	21%
16	24%
17	26%
18	28%
19	31%
20	34%
21	37%
22	41%
23	44%
24	48%
25	51%
26	55%
27	59%
28	63%
29	67%
30	71%
31	75%
